# Scaling up community-based health insurance in Ethiopia: a qualitative study of the benefits and challenges

**DOI:** 10.1186/s12913-022-07889-4

**Published:** 2022-04-10

**Authors:** Addis Kassahun Mulat, Wenhui Mao, Ipchita Bharali, Rahel Belete Balkew, Gavin Yamey

**Affiliations:** 1 Kilimanjaro Consulting, 341 Code1110 Addis Ababa, Ethiopia; 2 The Center for Policy Impact in Global Health, Duke Global Health Institute, Duke University, 310 Trent Dr, Durham, NC 27705 USA

**Keywords:** Health financing, Health insurance, Community-based health insurance, Ethiopia

## Abstract

**Background:**

Ethiopia has achieved impressive improvements in health outcomes and economic growth in the last decade but its total health spending is among the lowest in Africa. Ethiopia launched a Community-Based Health Insurance (CBHI) scheme in 2011 with a vision of reaching 80% of districts and 80% of its population by 2020. This study aimed to identify early achievements in scaling up CBHI and the challenges of such scale-up.

**Methods:**

We interviewed 18 stakeholders working on health financing and health insurance in Ethiopia, using a semi-structured interview guide. All interviews were conducted in English and transcribed for analysis. We performed direct content analysis of the interview transcripts to identify key informants’ views on the achievements of, and challenges in, the scale-up of CBHI.

**Results:**

Implementation of CBHI in Ethiopia took advantage of two key “policy windows”—global efforts towards universal health coverage and domestic resource mobilization to prepare countries for their transition away from donor assistance for health. CBHI received strong political support and early pilots helped to inform the process of scaling up the scheme. CBHI has helped to mobilize community engagement and resources, improve access to and use of health services, provide financial protection, and empower women.

**Conclusion:**

Gradually increasing risk pooling would improve the financial sustainability of CBHI. Improving health service quality and the availability of medicines should be the priority to increase and sustain population coverage. Engaging different stakeholders, including healthcare providers, lower level policy makers, and the private sector, would mobilize more resources for the development of CBHI. Training for operational staff and a strong health information system would improve the implementation of CBHI and provide evidence to inform better decision-making.

**Supplementary Information:**

The online version contains supplementary material available at 10.1186/s12913-022-07889-4.

## Introduction

Universal health coverage (UHC) aims to ensure people have access to the high quality health care that they need when they need it, without suffering financial hardship. In recent years, the UHC movement has gained global momentum [1]. However, most Sub-Saharan African countries have found it challenging to raise revenues for financing the delivery of an essential package of health services. In the face of this challenge, there has been growing interest in Social Health Insurance (SHI) and Community-Based Health Insurance (CBHI), as seen by the launch of SHI and CBHI schemes in Ethiopia, Ghana, Kenya, Lesotho, Nigeria, Rwanda, South Africa, Swaziland, Tanzania, Uganda, Zambia and Zimbabwe [2].

Ethiopia is one of the countries that spends the least on healthcare services in Africa. In 2016, health expenditure was only 1.4% of gross domestic product (GDP) [3]. National health financing indicators showed that only 11.1% of the total government budget was allocated to health in 2014 [4], below the 15% threshold target of the Abuja Declaration [5]. The public sector dominates the provision of health care services, with nearly 75% of hospitals run by the ministry of health or other government agencies [6, 7]. Public health care facilities provide healthcare services at nominal prices or free of charge depending on a patient’s ability to pay [6]. However, access to healthcare remains a barrier to reaching UHC in Ethiopia, especially in rural areas [8].

Ethiopia has shown improvements in health outcomes over the last decade. For example, the under-five mortality rate fell from 86.7 to 50.7 per 1000 live births between 2009 and 2019 [9]. The country has also experienced sharp economic growth in the last decade [10]. However, despite impressive gains in recent years, use of health services in Ethiopia remains low. The rate of outpatient visits per capita stands at less than 0.48 annually [10], far below the World Health Organization’s suggested rate of 1.0. In 2013, only 62% of individuals in Ethiopia reported seeking health services when they fell ill [11]. Out of pocket payment (OOP) accounted for about 34% of total health expenditure in 2017, indicating the heavy financial burden on households of paying for health care [11]. OOP also has a detrimental impact on use of services. In one study, over 40% of individuals not seeking healthcare when they were ill cited financial limitations as the primary reason [12].

To accelerate progress towards UHC, the government of Ethiopia piloted CBHI in 13 woredas (districts) between 2011 and 2013. CBHI is a voluntarily insurance scheme. The financial contribution during the pilot stage was 180 Ethiopian Birr (ETB) per household annually (10.4 US$ in 2011 US dollars). However, the members’ contributions varied among the pilot districts, ranging from 34.4 ETB to 132 ETB annually [13]. The CBHI benefits package includes all family health services and curative care (inpatient services, outpatient services and acute illnesses), which are part of Ethiopia’s essential health package [14]. Based on the encouraging results of CBHI pilots, the government scaled up the scheme to over 350 woredas in 2017. As a result, more than 14.5 million people had health insurance coverage through CBHI in 2017. The government then planned to expand CBHI to 80% of woredas and 80% of the population by 2020 [4].

In 2009, De Allegri and colleagues reported that enrolment rates for CBHI were less than 10% in sub-Saharan Africa countries [15]. In comparison, the Ethiopian scheme enrollment rate was over 50% on average, but coverage varied across the pilot regions. While the Amhara region recorded a 68% coverage rate, the highest rate among the four regions, coverage in the Tigray region was the lowest at 49%. Some of the factors that led to such variations in coverage of across the pilot regions included commitment from the local administration, waiting times at the facilities, and delays in renewing CBHI [16]. A USAID report found that the quality of service was poor at some CBHI health facilities and there were gaps in human resources, shortages of drugs and medical equipment, power outages and poor water supply [17]. Thirty nine percent of non-members raised concerns about the affordability of premiums and the registration fee. Combined with the 16% of non-members taking the “wait and see” approach, this suggested that the fees or premiums were too high to be affordable. Fiscal space for health from public sources needed to be expanded to ensure full coverage by CBHI of indigents (poor and very poor people) and to ensure the financial viability of the scheme [18].

After CBHI pilots were launched in 2011, several studies explored the willingness of people to join CBHI and the satisfaction of those who joined [13, 19–21]. Service provision, wait times, and procedures to get reimbursement from CBHI were associated with both willingness to enroll in CBHI and the satisfaction with CBHI [22, 23].

After 2016, a number of studies assessed the implementation of CBHI and its impact on service use, financial burden, quality and equity [24–32]. Abazinab et al. found that the public sector, the main provider for CBHI beneficiaries, was short of qualified personnel and laboratory services [24]. Mekonen et al. observed that CBHI provided significant financial protection from catastrophic health expenditure in northeast Ethiopia [29]. Lavers conducted a study on the political drivers of the adoption and evolution of state health insurance in Ethiopia [33]. Lavers’ study showed that while political commitment to self-reliance and long-term development helped to expand UHC and CBHI in Ethiopia, fragmentation and cross-subsidization to improve financial and administrative sustainability needed further attention.

While these studies identified gaps in and achievements of CBHI, little is known about the factors that contributed to the success of CBHI in Ethiopia and the potential barriers that may impede further achievements. This study therefore aimed to identify success in and challenges for scaling up CBHI.

## Methods

We used a qualitative design, which can be used to assess the opinions of policy makers, groups and organizations involved in the policy process and also affected by the policies [34, 35]. We used this design to explore how policy makers in Ethiopia understand the achievements and challenges of CBHI.

### Sampling technique

We used a purposive sampling technique to recruit key informants who had sufficient knowledge and expertise about the scale-up of CBHI. We conducted a desk-based review to identify key policy makers and stakeholders who directly and indirectly participated in the health financing and health insurance policy process in Ethiopia. Additional interviewees were identified through snowballing. We requested the participation of interviewees via telephone. Follow-up contact was attempted for participants who did not initially respond. We conducted interviews until theoretical saturation was reached.

### Data collection

We conducted semi-structured interviews with 18 key informants (KIs). The interview guide was developed based on the initial literature review and discussion among the authors. We pilot tested the interview guide with two interviewees and, based on the pilot testing, we then made minor modifications to the interview guide (the piloted interviews have been included in the analysis). The interview guide and the type of organization that the KIs are affiliated with (e.g. multilateral donor, bilateral donor, Ethiopian ministry of health, Ethiopian ministry of finance, insurance agency) are shown in Appendices 1 and 2 (any identifying features have been removed, to protect confidentiality). The participants were selected from national level government organizations (six KIs), multilateral and bilateral development partners (five KIs), non-government organizations (NGOs) (six KIs), and the private sector (one KI). One author (AKM), an experienced health policy researcher, conducted all of the interviews in English via telephone. Interviews lasted an average of 45 min. All interviewees consented to be tape recorded. The recordings were transcribed by external consultants based in India, and coded for analysis using NVivo 11 software.

### Data analysis

We performed directed content analysis (also called deductive content analysis) of the interview transcripts to characterize key informants’ views on the challenges and good practices related to CBHI [36]. Two authors developed the code book based initially on the interview transcripts. They then coded six full interviews to test the code book (Appendix 3). Emerging themes derived from coding the transcripts were added into the code book. Three authors then coded all the interviews using the revised code book.

We organized the emerging themes into two categories: good practices for the implementation of CBHI (six themes) and major challenges for the scale-up of CBHI (six themes).

### Ethical considerations

The study protocol was reviewed by Duke University’s Institutional Review Board (IRB) and was determined as not being human subjects research, and thus it received a waiver from full IRB review. Written informed consent form was obtained from each interview participant ahead of the interview session and oral consent was obtained from all participants prior to the interview. If the participant consented to being audio-recorded, interviews were audio-recorded and de-identified. To protect key informants’ confidentiality, no identifying information about them has been included in this paper. Participants were not compensated for their participation in the study.

## Results

### Good practices for the implementation of CBHI

Six themes emerged related to good practices in implementing CBHI.

#### Political commitment to UHC and preparedness for future donor transitions

Two thirds of interviewees said that the global call for action around UHC had become the top health priority for the Ethiopian government, and had helped to catalyze the adoption of CBHI in Ethiopia. To achieve UHC for both the informal and formal sectors, said the KIs, there was increasing demand for new approaches to health financing and resource mobilization. After reviewing practices from other countries (especially African countries), the informants argued that these factors accelerated the push for health insurance to become the first policy tool choice to achieve UHC in Ethiopia.

*“As part of SDGs [the Sustainable Development Goals], the government wants to meet universal health coverage by 2030 and one of the means is through health insurance. So, this is among the reasons behind designing the health financing and health insurance strategy.” KI 07*

Half of the KIs said that another key factor driving the adoption of health insurance was the country’s impending transition away from development assistance for health and the growing realization among country stakeholders that alternate health financing sources would need to be identified. Half of the interviewees expressed concerns that donor financing would start decreasing over time, especially given Ethiopia’s rapid economic growth in recent years. Financing through health insurance, they argued, has provided additional resources to support the pathway towards self-sufficiency in Ethiopia and has become a key policy agenda item in the health sector.

*“government health financing in the health sector remains usually donor dependent; if you look at the most recent national health accounts it is still about 35% of the health sector [that] is financed by external donors. Of course, this is down from about 50%, three to four years’ back … so then the government sees health insurance as one of the mechanisms of a way out from donor dependency of the health sector”-KI 08*

#### Strong leadership and accountability, and wide engagement of stakeholders

Most interviewees (15 out of 18) agreed that CBHI was driven and implemented by strong political will and support, and said that commitment and leadership at the federal level helped to mobilize resources and improve accountability of different government agencies. At the top level, explained the KIs, it also provides clear directions to enhance the engagement of the private sector and of NGOs.

*“I think, first of all, that the Ethiopian government is very strong in terms of political leadership which is in my humble opinion, one of the most important things if not the most important thing to have a successful health financing reform … generally, they can be quite directive in how they want to have NGOs and donors work within the country.”- KI 05*

KIs described how the strong political commitment at the top levels of the Ministry of Finance, Ministry of Health and Ethiopian Health Insurance Agency (EHIA) also contributed to the development of CBHI. Additionally, other related stakeholders, including regional governments, development partners, the private sector, international and local NGOs, civil society organizations (CSOs), medical associations and academic institutions widely participated in the development of CBHI at different stages and in different ways. For example, development partners and NGOs provided technical assistance, while regional governments played an important role in adapting the framework issued by the federal government and mobilizing the community.

*“The federal ministry of health provided leadership support for the health insurance initiative. Donors and then the developing partners were also involved in the design and implementation process in the form of providing technical assistance and financial support for the implementation of the program. Of course, we have other local actors like the regional governments, zonal administrations, woreda administrations, health service providers. They have their own roles and responsibilities, particularly if you see the regional governments in the area of the community health insurance [that] involved the community. The role of the regional governments & woreda administrations were very high [important] like in community mobilization and developing and adapting the legal frameworks that is provided from the federal government. Yes, there are a number of non-governmental organizations that are supporting the government in implementing health insurance, mainly the international NGOs, for example WHO is providing support, UNICEF is providing support, the World Bank is providing capacity building support, Clinton Health Access Initiative is also providing technical & financial support, the Korean International Health by the Korean government they are also involved. The degree of involvement could vary but [they are] providing support for the government in addition to our project.” —KI 14*

#### Promoting resource mobilization and community participation

About half of respondents believed that by putting health care financing and health insurance at the top of its policy agenda, this in turn helped the government to mobilize resources for the health sector. On the demand side, government subsidies can be provided through insurance. Additionally, premiums collected by CBHI from individuals also join the overall funding pool, and CBHI re-allocates the funds to those who need health care. Compared with paying out-of-pocket payments at the point of care, KIs argued that the pooling and financial risk protection provided by CBHI should improve access to and affordability of health care.

*“The objective of the health insurance is to generate additional finance for the health sector because we have already identified insufficiency of financing as one of the major problems of the quality compromised in Ethiopia. So it was assumed that the health insurance will bring additional resource to the health care system which is one of the objectives.”-KI 09*

Beyond financial contributions, said the KIs, communities were involved in decision-making and governance of the health system, which was a unique opportunity to promote community engagement. The fact that communities started to make health service providers accountable was an important driver for quality improvement in service provision. So far, explained the KIs, exempted health services of CBHI are provided for free, which motivated the community to use the public facilities more often instead of paying out of pocket for services in private facilities. The increased service use by CBHI beneficiaries provided additional incentive to the providers to be responsive to the community’s increasing health demands and to improve quality of health services.

*“Before CBHI programs, health facilities were not functional during weekends especially on Saturday... After the launching of CBHI, some facilities made Saturday a regular working day so that people from the rural community would access health care services to insured CBHI program members. This shows that the health facilities have become responsive for the need of the community.”- KI 15*

#### Improved access and service use and positive impact on women’s empowerment

KIs argued that increased enrolment in CBHI has contributed to changes in health seeking behaviors. Most KIs stressed that CBHI increased access to and use of both inpatient and outpatient services.

*“In those places [woredas/districts] where the health insurance scheme is properly implemented with broad community awareness and participation, service utilization has increased. In general, as health-seeking behavior improved the health service utilization has improved in CBHI woredas.” -KI 02*

In addition to changes in health service use, most KIs said that the scheme promoted women’s empowerment by reducing their financial burden in accessing health care. The scheme provided additional support for women to seek health care when it is was needed, and also helped to raise women’s voices and concerns in demanding better services through community mobilization in decision-making.

*“Women [are] also empowered and able to use the services since they were not forced to pay during the service provision. In Ethiopia women are mostly depending on their husbands. They are raising some issues if there is a gap. It also enables them to ask the facilities for their rights, collectively and individually.”- KI 08*

#### Financial protection helping to address equity and poverty reduction

More than half of the KIs agreed that CBHI provided financial protection through risk sharing, prepayment, and subsidies to address equity and poverty reduction in the resource-poor settings. In particular, there has been a “fee waiver” for those who could not afford health services; the government has paid on behalf of the poor population. Health service expenditures exceeding 3000 birr to 4000 birr have been covered through CBHI, which meant that out-of-pocket payments and catastrophic health expenditures of CBHI beneficiaries were reduced. One-third of respondents held the opinion that CBHI had helped the government’s efforts on poverty reduction in the community.

*“While the scheme helped the government as an alternative domestic resource mobilization platform, it helps the members of the insurance scheme to access and utilize health care services without too much worrying about financial expenses … So far, the scheme managed to reduce the out of pocket spending of the members though it is marginal.”- KI 12*

*“[On] equity, in areas where the woredas are committed to pay for the poor, it [CBHI] really had protected the poor. In woredas where there is almost universal health coverage, it [CBHI] is really bringing equity [in] utilization of services.” -KI 03*

#### Learning process from the pilot and from other countries

Many stakeholders (7 out of 18) believed that CBHI benefitted from the adoption of lessons learnt from the pilot program and from other country experiences. Before the introduction of CBHI, the Ethiopian government learned from CBHI schemes in other countries, including Rwanda and Ghana. CBHI pilots, which were only initiated in 13 districts, were manageable, and the assessment and feedback on the pilot helped to scale up CBHI to more woredas.

*“Implementation of the pilot first of course, CBHI scheme was really good, was very manageable because we started very small, we started with 12 districts, and then added more, so it was really manageable and it was I think a wise approach to start with [a] few districts and because it worked. There was a strong commitment from those selected districts. The government, regions, and district were happy to scale it up.” -KI 04*

### Major challenges for scaling up CBHI

Despite the good practices and successes of CBHI identified by interview respondents, KIs also identified a number of challenges that will need the attention of relevant bodies if CBHI is to provide UHC in Ethiopia.

#### Limited risk pooling and financial sustainability

More than two thirds of KIs were concerned that financial sustainability would be the most challenging issue for the further development of CBHI in Ethiopia. CBHI, by its nature, is implemented at the woreda level and beneficiaries join the scheme voluntarily. The voluntary nature of enrolment leads to a fragmented financing mechanism with limited risk pooling and reduced financial sustainability.

*“I think most of the challenges will be … .ensuring that everybody is enrolled, to ensure that the risk pools are actually able to be sustainable, that at some point, there is, maybe consolidation of the various woreda level schemes to ensure that they are more viable and financially strong. And I think these are some of the things that will be ironed out as times goes, there is more people enrolled in this scheme, they will see the benefit of enrolling in the scheme.”-KI 07*

Both KI 03 and KI 04 pointed out that some woredas have experienced “financial bankruptcy,” given the relatively small size of the funding pool. The factors that interviewees mentioned as being associated with financial unsustainability of CBHI included low coverage rates; coverage of mostly poor populations who cannot afford the fee and premium; and coverage with high numbers of people who use health services intensively. To improve the financial sustainability of CBHI, five KIs suggested that Ethiopia should consider increasing the level of risk pooling to improve cross-subsidization, while another four KIs argued that mandatory participation in CBHI should be considered. These options were also being discussed within the government.

*“We need to establish a pooling system to influence cross subsidization between woredas and also between regions”-KI 02*

*“From the very beginning, CBHI was actually designed to be voluntary, but now I think there is a discussion that from our own experience and from other countries experience as well, the experience shows that CBHI is voluntary and the capacity of the scheme is challenging and there is also adverse effect of selection which actually compromises the capacity of the scheme and the objective of the scheme. That is actually being discussed currently, because there is no legal framework for CBHI and now I think the government or health insurance agency is developing a draft proclamation to make it compulsory, but still there is a debate and discussion that it should not be compulsory.”-KI 16*

KIs also mentioned government subsidies as another challenge for the financial sustainability of CBHI. The CBHI pilots received subsidies from the government in a variety of formats, including human resources, administrative support and premium support for poor populations. After scaling up, it is not clear who will cover the cost of subsidies and provide administrative personnel to all the regions implementing CBHI.

*“The costs, the government has to subsidize since CBHI scheme has been implemented. Not only for CBHI, but also even for the SHI as well as because currently the public health centers are highly subsidized [at] about 20%. So in CBHI, the salary and all other costs actually the government decided that. When they decided to implement health insurance in Ethiopia, in the government of Ethiopia the higher-level officials or ministry of cabinet decided that the contribution of the beneficiaries will only be used for the services that they have got, so all the other costs will covered by the government.”- KI 11*

Finally, KIs argued that the operational plans for the benefit package, coverage and even premium had not been considered properly. Different elements of the CBHI scheme were not mapped out and connected with each other. For example, the scheme has not mapped actuarial projections to current premium levels and future claims pay outs, which may impact the scheme’s financial sustainability. While the premium was supposed to be affordable for most of the population, several stakeholders were concerned that the benefit package was “too generous” and cannot be afforded under current coverage rates and premium levels. Additionally, the moral hazard problem occurred whereby CBHI beneficiaries intended to use more services than the uninsured. Two stakeholders also mentioned this as another factor that could endanger the sustainability of CBHI.

*“CBHI was a very good model of bringing all [different levels of government and beneficiaries] together but what I found is operationally it has not [been] designed well yet. It is [at] a very initial stage managing the operation part of the scheme because how we are going to reach out to people, how the premium is going to be collected, how providers are to be paid … how the Ethiopian health insurance agency will treat between the public and private sector … I think that is something where I see a gap in EHI [Ethiopian Health Insurance] agency.”-KI 01*

*“We have made a promise of a very extensive and elaborate package of services, which might not be feasible in real term [s] so, therefore, a lot of empirical analysis needs to be done.” KI-10*

#### Lack of legal framework and poor institutional accountability

Most KIs stated that the lack of a proclamation (legal framework) presents institutional and structural barriers to implementation of CBHI and it makes accountability challenging. A legal framework would clarify the institutional and structural arrangements for CBHI, which would help implementation; KIs argued that such a framework is urgently needed.

*“Okay, the first one is the legal framework. We need to have legal framework, we need have the proclamation approved before we scale it up because that legal framework will correct and will address some of the challenges like the pooling, the voluntarily vs compulsory kind of issues of debate and that will address actually through that proclamation and there is also discussion to build the pooling at regional level and that needs to be addressed through the legal framework.”–KI 16*

KIs argued that implementation of CBHI is hindered by the separation of regulatory bodies and implementers. The Ethiopian Health Insurance Agency (EHIA) was established at national level to lead the overall development of CBHI, with branch offices at regional level. However, the woreda administrators or woreda health offices are accountable for the establishment, operation and management of the CBHI. There is no effective coordination between EHIA and woreda, and KIs expressed their concern that this lack of coordination would be even worse after the scaling up of CBHI. Moreover, district-level management structures are costly to replicate across the country and potentially not effective. CBHI is managed at the woreda level, with three dedicated staff members, computers and other equipment installed in woreda health or administrative offices.

*“Currently we have a critical challenge: when the agency (the Ethiopian health insurance agency) was established to manage and to lead health insurance throughout the country, but if you see Community-Based Health Insurance scheme, the scheme was established at woreda level and in some woredas, they are accountable for woreda administrators and in some regions, they are accountable for woreda health offices. So, there is no direct accountability line between the woreda and the branch office of the agency (the Ethiopian health insurance agency and its branches).” -KI 02*

#### Weak health service delivery system and limited engagement with private sector

Most KIs (15 out of 18) worried that the health service delivery system is not ready to provide proper care for CBHI beneficiaries. The current management, said the KIs, is unable to hold providers accountable for providing cost-effective, quality care and services are reimbursed on a fee for service basis without checks in place to control costs or quality. Many facilities provide low quality services and providers differ in their readiness to deliver quality care due to problems in staffing, medicines, laboratory facilities, reception, and outpatient services.

*“In terms of providers of services, the major area of concern is readiness of facilities, which is beyond the capacity of the providers. When we say readiness, it is infrastructure. Some of them do not have power, some of them do not have electricity, so the ability of the facilities to provide respectful care, responsive care, sometimes is limited.”-KI 03*

Most respondents raised weak supply chain management as one of the root causes of poor quality of services. The supply side constraints, they argued, in the areas of pharmaceutical and medical supplies procurement, transportation and distribution need much attention. Additionally, the implementation of CBHI has raised the expectations of the population in terms of better availability of services, especially access to pharmaceuticals.

*“The other [problem] is the availability of medicines. Medicines availability has highly improved in Ethiopia but still there is inequitability in Addis Ababa: you can find a lot of medicines and public facilities having majority of pre-essential drugs that are expected to be availed in the facilities based on the regulatory agencies standard guideline, but you can rarely find [these medicines] when you go to the health facilities. The availability of medicines is scarcely available.” -KI 09*

Eight KIs explained that while engagement with the private sector was encouraged and implemented at the federal level, this practice was not reflected at the regional level. CBHI is often affiliated with woredas and/or regional health bureaus at the regional level, making the public sector the dominant service provider for CBHI beneficiaries. Due to a lack of policies supporting public and private sector collaboration, private providers considered CBHI as a “threat” because CBHI has promoted service use at public sector facilities. Private providers were also excluded from the decision-making process of CBHI. The limited engagement from the private sector at the regional level further reduced the competition between public and private provision.

*“ … there is not enough competition in the health sector as I said before, so they [CBHI] have mentioned that the public facilities are included in the system and the scheme has not even included the private sector. So, essentially, they [providers of CBHI, i.e., the public sector] are characterized by poor services for the customers”-KI 12*

Three KIs reported that health workers also complained about an increased workload after the implementation of CBHI and a lack of incentives for taking on additional responsibilities. Despite the increase in health service use due to the increase in enrolment and coverage of the health services under CBHI, health providers were still receiving the same payment without compensation for the increased workload.

*“One of the major unintended effects on CBHI probably is overload for health workers... I think work overloads were not expected, particularly in good facilities. Although the health providers think that they are overloaded and hence should get incentives, the government insisted that this is the health provider’s normal duty … “KI 03*

All these factors, said the KIs, led to poor quality of health services or limited availability of medicines or medical supplies. Two interviewees pointed out that if these issues are not handled properly, CBHI beneficiaries would abandon the scheme.

*“If the people [CBHI beneficiaries] are paid for the services, they [beneficiaries] are serious about the premium and scheme. We have to be sure that the quality of services is available at the service delivery point.”-KI 15*

*“ … If you are a CBHI member and if services are not around, the insured community members started directly going to the districts administrators and say, “I am paying my money, where is the doctor? Where is the health provider? ”-KI 04*

#### Absence of political commitment at the sub-national level

Over half of informants stated that although political support for CBHI was very high at the federal level, there were significant variations in political commitment between regions and woredas. Even though representatives from woredas were invited to the discussion on CBHI, in most cases the level of engagement was low. Additionally, collection of CBHI premiums was regarded as a “demanding” task by the woredas, and without delegated efforts from the woreda level administration, resources at community level could not be fully mobilized and posed a major bottleneck for the scaling up of CBHI.

*“They [woreda representatives] are invited to discuss on sector strategies. I mean they don’t actively engage although there were many invitations so as to get all the partners to be engaged in the discussion, we have to invite them twice, maybe sometimes you have to invite them more. So, they are invited by the Ethiopian health ministry but actually honestly they are not engaged.”-KI 11*

*“Leadership commitments at all levels including the regional level, have been a challenge. There is a good commitment at federal level, but when you go to the woreda level, there is a difference, significant difference between woredas. Some of the woredas are able to achieve 100% [coverage], but there are woredas who only gained 20% coverage because of leadership commitment, so that they [woredas] are important.” -KI 02*

#### Constrained implementation capacity, especially at the woreda level

Half of the KIs argued that lack of human and institutional capacity of the regional health and finance bureaus and woredas was a barrier to scaling up CBHI. The Ethiopian health insurance system needs further capacity in terms of its human resources, clear management structure and system building. Citing examples of how investments in other countries significantly contributed to health sector improvement, most respondents felt that capacity building and training were crucial to improving the implementation of CBHI in Ethiopia.

*“ … The capacity challenge is not only in terms of skills but also quantity of the required staffing at the lower level. Supporting capacity building of the government at woreda level is particularly important. For example, there is only one person who is responsible for the kebele [neighborhood] working as a focal person for the scheme who is usually kebele manager who is only supported by the kebele’s community. He is in charge of all the activities in that particular kebele … ”-KI 13*

Respondents mentioned that there is a lack of human resource capacity at lower levels, including limited ability at the woreda level to manage risks through clinical and financial auditing and through cash management. Providers also faced challenges in processing claims. In addition, variations in the commitment of the local management officials raised concerns about the sustainability of the schemes, as CBHI implementation depends heavily on the woreda administrative staff.

*“Opening [CBHI] branch offices is not difficult, you can open branch offices, you can install the required infrastructure and so on, but if the country doesn’t have adequately trained officials who can manage, administer, and lead the process … .The health service will not be able to catch up all expectations of the community” -KI 04*

To address the capacity issue, many stakeholders advocated for changes in the capacity building system such as the system of training and transferring skills from the federal to the lower levels. For example, professionals should have the chance to attend various trainings and learning opportunities to incorporate lessons and experiences of other countries in their health financing and health insurance practices.

*“ … There needs to be an in-built system at all levels, especially from the federal level, to share the experiences they learnt from abroad and really ramping up its effort towards training all the way down to the regional and woreda levels. I think the big challenge is down at the woreda and kebele levels. So, the capacity building is usually very difficult to maintain and constantly sustain it. But it is important to cascade that information to the lower levels to bring comprehensive changes in the system.”- KI 06*

#### Weak information systems to support daily operation and informed decision

Nearly half of all interviewees (7 out of 18) raised concerns about the health information system. The dependence on manual systems for the operations of CBHI led to reduced efficiency in core operations such as enrollment, claims management, and auditing. Digitalization and information technology (IT) systems will need to be adopted to manage the huge volumes of claims and to improve service provision if the scheme is to be scaled up to the national level. KIs argued that the lack of good information systems is a challenge at the central, regional and woreda levels for tracking progress and addressing problems in CBHI in a timely, transparent and reliable manner.

*“ … The Government needs to work closely with the relevant stakeholders in order develop the information system so that they collect accurate and reliable data related to the premium collection, claims management and disbursements related to health insurance at all levels and analyse them for better decision-making.” -KI 05*

## Discussion

Our qualitative study with key informants from Ethiopia identified several good practices and successes in scaling up CBHI, but also several challenges. The key informants argued that the implementation of CBHI in Ethiopia was a result of a political drive towards self-reliance and UHC, and was informed by lessons from other countries and the results of the domestic pilot scheme. Positive impacts of CBHI, including resource mobilization, improved service use and financial risk protection, have in turn encouraged enrolment into the CBHI scheme. However, the respondents also identified major barriers that need to be addressed in order to successfully scale up CBHI nationwide.

Stakeholders in our study suggested that Ethiopia had done well to adopt three “good practices” in the initial phase of its CBHI implementation. First, global efforts towards UHC and concerns about future donor exits from Ethiopia pushed the government of Ethiopia to seek alternative health financing approaches and to provide a strong political commitment to CBHI. The two “policy windows” of UHC and donor exits were fully leveraged and transformed into strong political commitment, and helped to create policy momentum and support for mobilizing resources for CBHI in Ethiopia.

Second, Ethiopia’s CBHI drew lessons from experiences of other countries and widely engaged with stakeholders for additional technical support. The lessons from the initial CBHI pilots in select districts also helped to improve the design and further development of the program.

Third, Ethiopia managed to mobilize domestic resources through CBHI, which creates a pathway to achieve UHC goals and objectives with broader scale-up efforts. The increasing community participation improved the responsiveness of the health delivery system and improved health service use. The scheme also had a positive impact on women’s health seeking behaviour and their participation in decision making. This can lead to empowerment of women and improved financial protection, which in turn can help to improve community support for scaling up the scheme. Stakeholders in our qualitative study also argued that CBHI has increased service use and reduced the financial burden for CBHI beneficiaries. Other studies, including those with quantitative designs, have had similar findings. For example, in a cross-sectional study, Atnafu and colleagues observed that CBHI beneficiaries had almost double the service use of the uninsured population—the rate of healthcare use was 50.5% in insured households versus 29.3% in uninsured households [25]. In another cross-sectional study, Mekonen et al. found that less than 5% of households with CBHI experienced catastrophic health expenditure compared with over 15% of uninsured families [29].

However, our study also found that there are many challenges that need to be addressed to improve the sustainability of CBHI. The key informants described three key obstacles. The first and most substantial challenge is the risk pooling mechanism. A fragmented fund pool is one of the most significant limitations of CBHI. The voluntary nature of CBHI is also inevitably threatened by adverse selection—sicker people are more likely to join the scheme, thus making it harder to achieve financial sustainability of the insurance fund. To address these challenges, it will be crucial to establish regional and national risk pooling mechanisms to increase cross-subsidization and to improve the capacity to share risk among various groups. Innovative solutions are needed to address adverse selection, such as encouraging family enrollment or even complementary insurance. A legal framework for CBHI should be adopted to give explicit guidance on the establishment of a pooling system to influence cross subsidization between woredas and between regions. This framework would also clearly guide what type of health structures are going to be implemented at woreda, zonal, regional, and national levels, and would clarify the roles and responsibilities of Federal and Regional Health Bureaus and local/woreda administrations with respect to health insurance schemes.

A second challenge that the KIs identified is the readiness of health facilities to be included in the CBHI scheme. Previous research has had similar findings. For example, Abazinab et al. conducted a facility-based cross-sectional study in Ethiopia’s Jimma zone [24]. They found that: “More than nine out of ten facilities did not fulfill the criteria for providing healthcare services for insurance beneficiaries and are not ready to provide general services according to the standard.” These findings echo the concerns expressed by stakeholders in our study.

KIs in our study provided insights on the factors that led to the poor performance of providers in the CBHI scheme, including the weak health system delivery system and lack of engagement with the private sector. Access to quality care is critical to expand and maintain enrollment of the population. Therefore, addressing poor quality should be the top priority for the government before scaling up the scheme to make sure all facilities have the required medicines, skilled professionals and good governance. This requirement calls for broader reform or policy changes in Ethiopia, including to ensure mobilization of the private sector and NGOs to improve their participation, especially at sub-national levels. A broader liberalization reform effort by the government is needed to enhance competition between the public and private sectors. Providing proper incentives to health providers should also be an important agenda to improve the overall responsiveness and quality of health services.

Third, the success of the CBHI scheme hinges on continued political commitment and engagement of governments at all levels. Given the weak domestic revenue mobilization capacity, the challenges created by the COVID-19 pandemic, and the socio-political conflicts in certain parts of the country, continued commitment and political will is going to be crucial to sustain the scheme. The Second Health Sector Transformation plan (HSTP II) for the period 2020–2025 reiterates Ethiopia’s commitment towards UHC. Among the key HSTP II strategic areas, the plan aims to increase financial protection through the CBHI scheme and aims to cover 80% of the informal sector in 85% of woredas by 2025, including the poor, women, and children. The HSTP II also emphasizes ‘woreda transformation’ to align national and regional priorities, and strengthen the capacity of woredas to achieve the country’s UHC goals [4]. Looking ahead, Ethiopia’s ambitious health sector agenda will need to be accompanied by strong reform, accountability, and a political movement to transform goals into actions and outcomes.

Finally, transition from a manual to an automated system is also necessary to improve the efficiency of the operation of CBHI and to inform improved decision making. The government should work closely with the relevant stakeholders in order to develop the information system to collect and analyze accurate and reliable data related to premium collection, claims management and disbursements at all levels for better decision-making.

While the scheme has yielded some good results, our study has also pointed to ways in which its performance can be improved. Recent studies of the implementation of Ethiopia’s CBHI and the satisfaction of enrollees suggest moderate satisfaction among beneficiaries with the scheme, with scheme awareness, premium payments, quality service provision, and scheme management being important determinants for improved provision [19, 30, 37]. A systematic review of the barriers to and enablers of CBHI in low- and middle-income countries identified family and socio-cultural factors, favorable political and policy environment, service accessibility, timing and amount of premiums, sustainable contributions and government budgets for successful enrollment and delivery [38]. These are echoed in the findings of our study, and need to be considered for further scale up and improved scheme performance and sustainability.

### Strengths and weakness of the study

One strength of our study is that is has been able to identify root factors that enable or impede the further improvement of CBHI in Ethiopia. It has also highlighted good practices identified through interviews with key stakeholders that provide a strong rationale to improve and build upon the early achievements to scale up CBHI. We interviewed a wide range of informants across different sectors and reached theoretical saturation, so it is likely that we captured most of the key views about CBHI among country policymakers.

Nevertheless, as with any qualitative study of this kind, our study also had a number of limitations—we highlight two in particular. All interviews were conducted in English, which is a widely spoken foreign language in Ethiopia. Language proficiency may have restricted the interviewees expressing their nuanced opinions, which in turn could have led us to having a limited picture of their full views. However, given how widespread spoken English is in Ethiopia, we believe language proficiency is unlikely to have had a discernible impact on our key findings and conclusions. Second, time, funding, and capacity constraints meant that we did not include subnational policy makers, implementers, service providers or CBHI beneficiaries as key informants. Therefore, our findings only captured the opinions of the national stakeholders who we interviewed; our study did not capture the views of subnational stakeholders. Further research, including interviewing a more diverse group of stakeholders is needed to fully explore issues around CBHI.

## Conclusions

The financial sustainability of CBHI will depend on efforts by the government to scale up domestic revenues and gradually increase the level of risk pooling. Improving health service quality and the availability of medicines should be the priority to reach a wider and sustained population coverage. Engaging different stakeholders, including healthcare providers, lower level policy makers, and the private sector, would mobilize more resources for the development of CBHI. Training for operational staff and a strong information system would improve the implementation of CBHI and provide evidence to inform decision.

## Supplementary Information

**Additional file 1.**

## Data Availability

To follow the IRB protocol for this study, neither interview records or transcripts will be available. However, we are happy to share an anonymized, de-identified summary of the interview transcripts. Please contact Dr. Gavin Yamey for details.
