# Supplementary material for: Scaling up community-based health insurance in Ethiopia: a qualitative study of the benefits and challenges
Source: BMC Health Serv Res. 2022 Apr 10;22:473. doi: 10.1186/s12913-022-07889-4 (PMC8994817; doi:10.1186/s12913-022-07889-4)
Supplement: Supplementary file 1 — Additional file 1. [file 12913_2022_7889_MOESM1_ESM.docx]

Emerging BENEFITS OF and challenges for scaling up community-based health insurance in ethiopia: a qualitative study

Addis Kassahun Mulat^1,2^*, Wenhui Mao^2*^, Ipchita Bharali^2^, Rahel Belete Balkew^1^, Gavin Yamey^2^

1 Kilimanjaro Consulting, 341 Code1110, Addis Ababa, Ethiopia

2 The Center for Policy Impact in Global Health, Duke Global Health Institute, Duke University. 310 Trent Dr, Durham, NC, 27705, USA.

*Contributed equally

**Corresponding to:**

Gavin Yamey MD MPH MA

310 Trent Dr, Durham, 27705, NC, USA

(919) 613-6221

[gavin.yamey@duke.edu](mailto:gavin.yamey@duke.edu)

## Appendix 1 INTERVIEW outlines

**INTERVIEWER READ: Hello, my name is [NAME] and I am [POSITION].** Thank you so much for having this telephone interview with me today and taking time. **Have you received the information sheet I sent you through your email?** As noted in the information sheet, **we will take all necessary steps to ensure your confidentiality**. The purpose of this interview is to get your opinions and views on the health financing and health insurance policies and strategies in Ethiopia. The information and data collected generated from this interview will be used for policy analysis. **If you agree to participate voluntarily,** this discussion will take about 45 minutes to an hour, I would like to learn from you about the policy which drives Community-Based Health Insurance (CBHI) and Social Health Insurance (SHI) schemes pilot project, expansion and possible scale up processes in the country. **Do you agree to participate voluntary? Is it OK to you to use Audio Recording so that I can focus on our interview discussion? 1) Ok 2) Not Ok**

Any questions before we begin 1. Yes 0. N0

1. Please could you tell me in which sector you are working?
2. Government at federal or regional Level c. Private sector
3. International or local non-governmental organization d. Multi-lateral agency
4. Civil Society Organization or Professional Associations f. Bilateral Agency g. Others
5. What is your role and level of your engagement in health financing and health insurance policy?
6. What did it make health insurance policy to become a policy agenda in the first place?

***Probe***: What were the aims and objectives of the health insurance policy in Ethiopia? ***(*Policy Content**

***Probe: How SMART are the HI objectives? (Policy Content*)**

1. Could you explain the ***background*** ***context*** under which the policy was initiated first? ***Hints***: It could be ***global, national or health sector situations*** that influence the policy options
2. Who were the main ***stakeholders/actors*** and their **roles during the Health Insurance policy design**? What were ***their levels of political influence*** in shaping the design?

***Probe:*** What were the ***main areas of differences*** among stakeholders involved in CBHI and SHI policy if you recall? Do you think still those differences exist currently?

***Probe:*** Do the opponents of the HI policy have the ***political or other powers of influence/ability to interfere*** with the adoption or implementation?

1. What do the **design and development, implementation and evaluation** of the Health Insurance policy look like? (***Policy Process***)

***Probe:*** What do stakeholders think of the proposed health insurance policy and the conditions surrounding adoption and implementation of this policy?

1. How do you see the coordination between the FMOH, EHIA and woreda/district administrations?

***Probe****:* What are the major **structural barriers** to the scheme for scale up into nationwide program?

1. What are the **major *outcomes/effects*** of the Health Financing and Health Insurance policies on the health services outcomes?

***Probe:*** How do you compare the ***intended outcomes*** of the health Insurance policy **with what it actually achieved?**

What are the **unintended positive** effects?

What are the **unintended negative** **effect**s of the HI policy?

1. ***What are the effects (intended or unintended) of the policy on different groups?***
2. How do you ***rate*** the health insurance policy impacts in terms of the following ***six dimensions***

***(Hint: What do stakeholders think of the proposed policy in terms of the following 6 dimensions?)*** : (**Policy Content**)

| **Dimensions** | **Rating** | | | | | | | **Do you have some possible sources of evidence you can mention, if possible?** |
| --- | --- | --- | --- | --- | --- | --- | --- | --- |
|  | **Low** | | **Moderate** | |  | **High** | |  |
|  | **Very Low** | **Low** | **Less Moderate** | **Moderate** |  | **High** | **Very**  **High** |  |
|  | **1** | **2** | **3** | **4** | **5** |  | 6 |  |
| **Effectiveness** |  |  |  |  |  |  |  |  |
| **Equity** |  |  |  |  |  |  |  |  |
| **Unintended effects** |  |  |  |  |  |  |  |  |
| **Cost** |  |  |  |  |  |  |  |  |
| **Acceptability** |  |  |  |  |  |  |  |  |
| **Feasibility** |  |  |  |  |  |  |  |  |

1. As you know the country is planning to integrate CBHI and SHI to make one national Health Insurance Scheme by 2020. What are the ***most important challenges*** that the schemes have faced in the expansion or scale up of the program? Tell us in terms of their level of significance

1^st^-------------------------------------------------------------------------------------------------------------------------

2^nd^ -----------------------------------------------------------------------------------------------------------------------

3^rd^ -----------------------------------------------------------------------------------------------------------------------

1. What are your ***major suggestions as solutions*** to address the challenges and improve the health insurance policy in the scale up of CBHI and SHI into National Health Insurance (NHI) Scheme for achieving better UHC?

1^st^-------------------------------------------------------------------------------------------------------------------------

2^nd^ -----------------------------------------------------------------------------------------------------------------------

3^rd^ -----------------------------------------------------------------------------------------------------------------------

***Probe:*** What **areas of the policy** you think need ***change*** for the country to **scale up CBHI and SHI into the National Health insurance** (NHIP)?

1. Do you have anything to add?
2. Who else do you recommend to be interviewed for this research?

## Appendix 2 List of interviewees

| **KII Code** | **Institution** |
| --- | --- |
| KII1 | International non-governmental organization (NGO) |
| KII2 | Health Insurance Agency, Ethiopia |
| KII3 | Ministry of health, Ethiopia |
| KII4 | Ministry of health, Ethiopia |
| KII5 | International NGO |
| KII6 | Ministry of health, Ethiopia |
| KII7 | Multilateral donor |
| KII8 | Bilateral donor |
| KII9 | Health Insurance Agency, Ethiopia |
| KII10 | Bilateral donor |
| KII11 | Health Insurance Agency, Ethiopia |
| KII12 | Academic institution |
| KII 13 | Ministry of health, Ethiopia |
| KII14 | International NGO |
| KII15 | Bilateral donor |
| KII16 | Multilateral donor |
| KII17 | International NGO |
| KII18 | Ministry of Finance and Economic Development, Ethiopia |
